# Fecal microbiota transplantation unveils sex-specific differences in a controlled cortical impact injury mouse model

**DOI:** 10.3389/fmicb.2023.1336537

**Published:** 2024-02-12

**Authors:** Tulasi Pasam, Manoj P. Dandekar

**Affiliations:** Department of Pharmacology and Toxicology, National Institute of Pharmaceutical Education and Research (NIPER), Hyderabad, India

**Keywords:** gut microbiome, fecal microbiota transplantation, traumatic brain injury, probiotics, controlled cortical impact

## Abstract

**Introduction:**

Contusion type of traumatic brain injury (TBI) is a major cause of locomotor disability and mortality worldwide. While post-TBI deleterious consequences are influenced by gender and gut dysbiosis, the sex-specific importance of commensal gut microbiota is underexplored after TBI. In this study, we investigated the impact of controlled cortical impact (CCI) injury on gut microbiota signature in a sex-specific manner in mice.

**Methods:**

We depleted the gut microflora of male and female C57BL/6 mice using antibiotic treatment. Thereafter, male mice were colonized by the gut microbiota of female mice and vice versa, employing the fecal microbiota transplantation (FMT) method. CCI surgery was executed using a stereotaxic impactor (Impact One™). For the 16S rRNA gene amplicon study, fecal boli of mice were collected at 3 days post-CCI (dpi).

**Results and discussion:**

CCI-operated male and female mice exhibited a significant alteration in the genera of *Akkermansia*, *Alistipes, Bacteroides, Clostridium, Lactobacillus, Prevotella*, *and Ruminococcus*. At the species level, less abundance of *Lactobacillus helveticus* and *Lactobacillus hamsteri* was observed in female mice, implicating the importance of sex-specific bacteriotherapy in CCI-induced neurological deficits. FMT from female donor mice to male mice displayed an increase in genera of *Alistipes*, *Lactobacillus*, and *Ruminococcus* and species of *Bacteroides acidifaciens* and *Ruminococcus gnavus*. Female FMT-recipient mice from male donors showed an upsurge in the genus *Lactobacillus* and species of *Lactobacillus helveticus*, *Lactobacillus hamsteri*, and *Prevotella copri*. These results suggest that the post-CCI neurological complications may be influenced by the differential gut microbiota perturbation in male and female mice.

## Graphical abstract

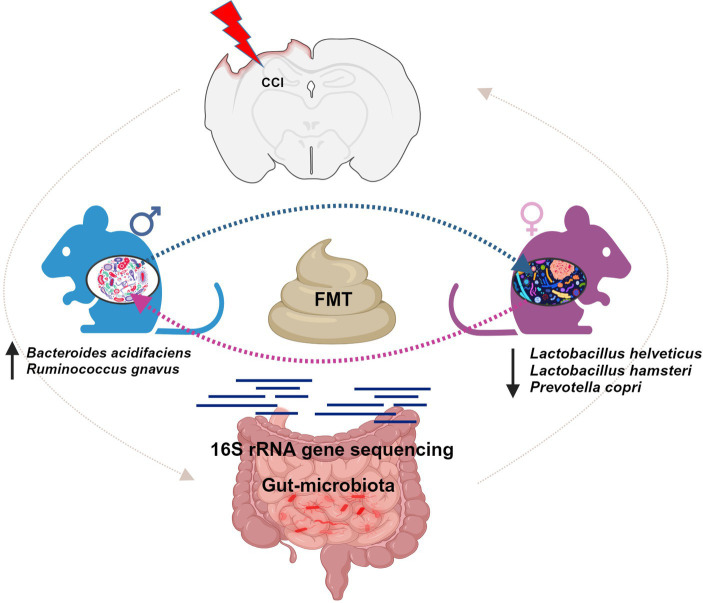

## 1 Introduction

Acute injuries linked with spinal cord and traumatic brain injury (TBI) as well as stroke pose several health problems, including neurological disabilities (Sun et al., 2018; Wang et al., 2018). TBI comprises a sudden mechanical injury to the brain by an external source (Coronado et al., 2005; Dewan et al., 2018). Each year, approximately 27–69 million people experience TBI incidence worldwide (Dewan et al., 2018; James et al., 2019). Experimental TBI in mice was reported to increase intestinal permeability and levels of endotoxins (Hang et al., 2003; Ma et al., 2017). Post-TBI gut microbiota perturbation was reported to negatively impact the recovery and lesion size (Nicholson et al., 2019). Indeed, growing literature documented the putative gut-brain crosstalk and the role of gut microbiota in the regulation of central nervous system (CNS) functions (Tan et al., 2011; Hazeldine et al., 2015; Treangen et al., 2018). The disturbed gut microbiota phenotypes have been evident in numerous neurological diseases such as autism, Alzheimer’s disease, depression, TBI, and Parkinson’s disease (Nicholson et al., 2019; Palepu and Dandekar, 2022; Rahman and Dandekar, 2023). Several clinical microbiome projects, investigating the connection of human gut microbiota with brain functionality and disease severity, are underway (Qin et al., 2010; The Human Microbiome Project Consortium, 2012a,b). It has also been suggested that the disruption of gut–brain crosstalk in the aftermath of TBI may affect the functioning of the central and enteric nervous system (Tan et al., 2011; Evans et al., 2015; Hazeldine et al., 2015; Sundman et al., 2017; Treangen et al., 2018; Ibrahim et al., 2022). The replenishment of healthy microbiota using probiotics confers positive impacts on several brain functions (Bienenstock et al., 2015; Sirisinha, 2016). For instance, the restoration of disrupted gut microbiota using probiotics was found useful in the management of dementia, depression, stroke, and inflammatory bowel diseases (Gao et al., 2017; Lye et al., 2018; Palepu and Dandekar, 2022; Rahman and Dandekar, 2023). Thus, perturbation of the gut microbiota may be directly linked to the progression of the disease (Louis, 2012; Sampson et al., 2016; Zheng et al., 2016; Jiang et al., 2017; Yuan et al., 2018).

In the acute CCI model, Treangen and colleagues reported a decreased abundance of the genus *Lactobacillus and Ruminococcus* and an elevation in *Eubacterium and Marvinbryantia* at 1 day post-injury (dpi) (Treangen et al., 2018). Nicholson et al. also showed that TBI may also results in gut microbiota dysbiosis, which was correlated with the lesion volume (Nicholson et al., 2019). Moreover, other TBI studies also reported changes in *Lactobacillus*, *Romboutsia,* and *Turicibacter* microbial genera following the injury (Kang et al., 2019; Koopman et al., 2019; Li et al., 2019; Du et al., 2021). Furthermore, probiotic treatment has shown potential benefits in animal models of spinal injuries, TBI, and stroke (Akhoundzadeh et al., 2018; Ma et al., 2019; Devi et al., 2021; Savigamin et al., 2022). In clinical settings, supplementation of *Lactobacillus*-rich probiotics showed improvement in TBI patients by modifying gut dysfunction (Tan et al., 2011). Additionally, replenishment of gut microbiota is also modified by fecal engraftment techniques improving neurological performance and shifting the pathobiome to healthy microbiota (Van Nood et al., 2013; Kelly et al., 2016; He et al., 2017). Taking into consideration the two-way connection between the gut microbiota and the brain, we intended to determine whether the gut microbiota is influenced by CCI.

Recent research has increasingly highlighted the sex-specific differences in TBI, encompassing variations in prevalence, disease severity, rates of emergency hospitalizations, and mortality rates (Faul and Coronado, 2015; Cox et al., 2019). Furthermore, disparities in the constitution of the gut microbiome between male and female mice have been associated with the development of chronic diseases (Markle et al., 2013; Fransen et al., 2017). Notably, male mice tend to exhibit a higher abundance of genera like *Alistipes*, *Rikenella*, and *Porphyromonadaceae* in their gut microbiota, while female mice show increased levels of *Akkermansia* and *Lactobacillus* (Fransen et al., 2017). These distinctions in the gut microbiome composition potentially contribute to divergent responses to TBI severity and other associated comorbidities in male and female mice (Deitch et al., 2007; McGlade et al., 2015; Lavoie et al., 2017). Interestingly, there is an observed trend of greater resilience to post-traumatic consequences among women (Spychala et al., 2017). However, it is of crucial concern to note that the existing clinical evidence regarding sex-specific outcomes following TBI is still a subject of debate and controversy (Caplan et al., 2017).

This study aims to examine the sex-specific changes in gut microbiota following CCI surgery in both male and female mice. Moreover, the opposite-sex FMT approach was adopted, i.e., fecal slurry of male mice was transplanted into female mice and vice versa. The gut microbiota signature was evaluated from the samples collected at 3 dpi using the 16S rRNA v3 + v4 gene sequencing method. This study focused on the gut microbiome profiling, in particular, to identify the bacterial level abundance. Multiple studies have drawn observations stating that TBI-induced microbiome alterations often lead to a neuroinflammatory cascade ending up with secondary TBI (Ma et al., 2017; Nicholson et al., 2019).

## 2 Materials and methods

### 2.1 Animal housing, grouping, and CCI surgery

Adult, male and female, C57BL/6 mice (8–12 weeks) weighing 20–30 g were housed in polypropylene cages with free access to water and regular laboratory chow diet. The experimental and holding rooms were meticulously controlled to ensure a stable environment, with temperature maintained at 25 ± 2°C, humidity at 55 ± 5%, and an uninterrupted 12–12 h light–dark cycle. All experiments were conducted in compliance with the rules and guidelines of the Institutional Animal Ethics Committee of the NIPER, Hyderabad (Approval number: NIP/10/2020/PC/377).

Experimental mice were divided into six groups (*n* = 4): Group 1: Sham_M (Sham-operated male mice), Group 2: Sham_F (Sham-operated female mice), Group 3: CCI_M (male mice subjected to CCI surgery), Group 4: CCI_F (female mice subjected to CCI surgery), Group 5: CCI_M → F (female mice received FMT from male donor mice and then underwent CCI surgery), and Group 6: CCI_F → M (male mice received FMT from female donor mice and then underwent CCI surgery).

The surgical procedure of CCI has already been standardized in our laboratory (Rahman et al., 2022; Tentu et al., 2023). This procedure creates a highly reproducible brain injury that replicates the condition of a cortical contusion in humans. Briefly, mice were anesthetized with a mixture of isoflurane (4% for induction and 1–2% for maintenance) in 95% oxygen. The individual mouse was then placed on the stereotaxic apparatus (Stoelting) and secured with the use of a palate bar, nose clamp, and ear bars on both sides. CCI surgery utilizes a piston to apply controlled mechanical force through a surgical craniotomy directly to the exposed dura, typically 3–4 mm in size. Midway between bregma and lambda craniotomy was performed in the left parietal bone with the medial edge 0.5 mm lateral to the midline leaving the dura intact. The impact was delivered using a stereotaxic impactor (ImpactOneTM, Leica) at a velocity of 5 m/s with a 3 mm wide impactor, 0.1 s dwell time, and 1.5 mm deep followed by re-placing the skull flap on the exposed region to close the incision. A bead sterilizer (Steri 250) was used for the sterilization of surgicals across the animals during surgery. Sham mice of both sexes (Sham_M and Sham_F) also underwent the same surgical procedure, except impact was not delivered.

### 2.2 Fecal microbiota transplantation procedure

Fecal boli of male and female donor mice residing in different cages were collected at 3 dpi and immediately kept at −80°C for further use. Fecal samples of male and female donor groups were pooled and homogenized for 5 min using chilled phosphate buffer (120 mg feces/1 mL buffer) to make a paste-like consistency. Then, followed by centrifugation at 800x g for 3 min. Finally, the obtained supernatant was aliquoted in sterile tubes kept at −80°C for further use (Spychala et al., 2018).

**Figure 1:**
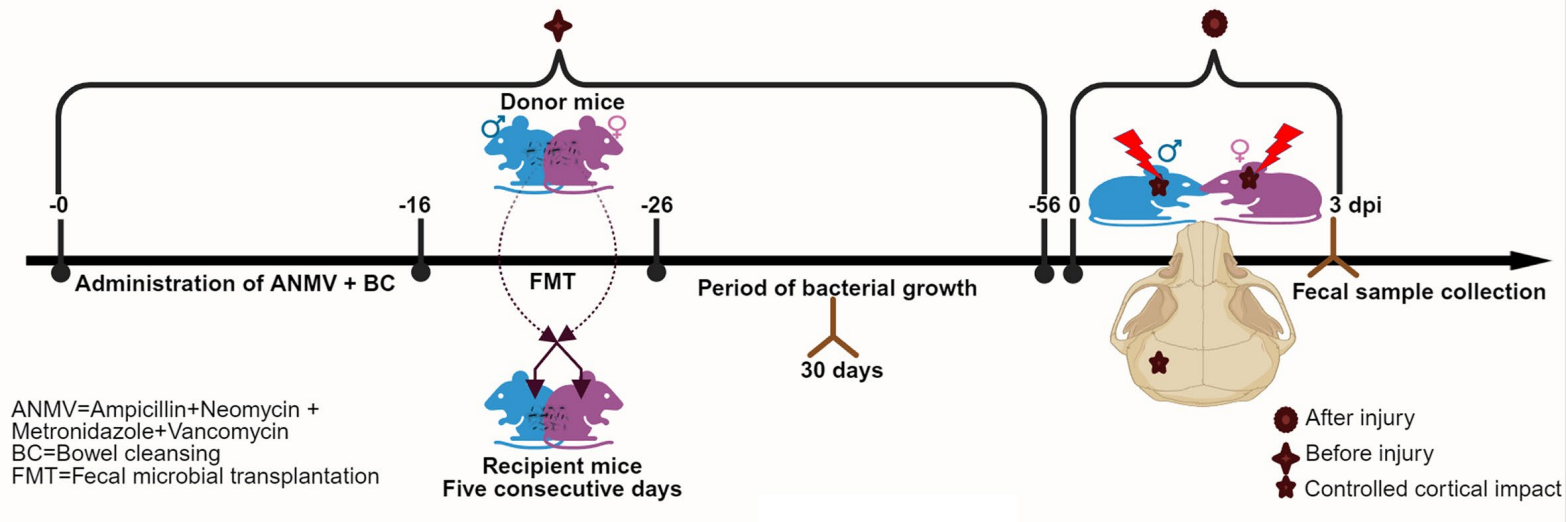
An *in-vivo* study design showing the timeline for antibiotic treatment, FMT, CCI, and fecal sample collection in mice.

Male and female mice were prepared for the FMT procedure by peroral administration of ampicillin sulfate (A), neomycin sulfate (N), metronidazole (M), and vancomycin hydrochloride (V) for 14 days (Morgun et al., 2015; Leclercq et al., 2020). Post-antibiotic treatment, the bowel cleansing (BC) was done using a solution of PEG3350, sodium bicarbonate, sodium sulfate, sodium chloride, and potassium chloride (Le Roy et al., 2019). Followed by the administration of prepared microbiota slurry by oral gavage (200 μL) to each recipient for 5 consecutive days once daily (Figure 1), FMT was carried out by administering a fecal slurry of male mice to the female recipient (CCI_M → F) and a fecal slurry of female mice to the male recipient (CCI_F → M). As described in Section 2.1, CCI surgery was adopted 30 days post-FMT.

### 2.3 Fecal collection for 16S rRNA gene amplicon study

Fecal samples were individually pooled from both male and female mice, which were housed temporarily in sterile cages without bedding on 3 dpi. These samples were then collected in sterile tubes and evenly distributed among all the experimental groups for further extraction steps.

### 2.4 DNA extraction

The genomic bacterial DNA was extracted from fecal samples using a QIAamp PowerFecal Pro DNA Kit (Qiagen catalog no: 51804). The procedure for the extraction of DNA from rodent fecal samples has been standardized and published in our earlier studies (Rahman et al., 2023). Briefly, 200–250 mg of fecal sample was added to the PowerBead Pro Tubes containing 800 μL of CD1 solution and then homogenized using a FastPrep-24™ bead grinder (MP Biomedicals, United States) followed by centrifugation at 15,000 × g for 1 min. Then, 200 μL of CD2 solution was added to the supernatant and centrifuged for 1 min. The supernatant was again transferred to a new tube containing 600 μL of CD3 solution, passed through the MB spin column, and subsequently washed by 500 μL of EA and C5 solution. The column was then placed into a new tube, and 50–100 μL of solution C6 was added to the center of the white filter membrane. The DNA was collected after centrifugation at 15,000 × g for 1 min.

### 2.5 Library preparation

A total of 20 ng of DNA was used for the KAPA HiFi HotStart ReadyMix PCR Kit (Catalog: 0370, KAPA BIOSYSTEMS) and hypervariable regions (V3-V4) in the 16S gene amplicon assay (Amplicon et al., 2013). The process involves DNA (20 ng), primers of 100 nM concentration (341F and 785R), and KAPA HiFi HotStart ReadyMix (2X). PCR steps include denaturation for 5 min at 95°C, 20 cycles for 30 s at 95°C, accompanied by 45 s at 55°C, and for 30 s at 72°C, then followed by final extension for 7 min at 72°C and holding at 40°C. To confirm the amplification results, the PCR sample (3 μL) was loaded on agarose gel (2%) and the bands were notified at ~456 bp. To eliminate unused primers, cleansing of PCR entities was carried out (Catalog: A63881, Beckman Coulter) using XP beads 0.9X AMPure. Furthermore, eight cycles were performed for the purified PCR contents (4 μL) with a unique P7 and P5 barcoding process. The last set of PCR products underwent a secondary purification step with 0.9X AMPure XP beads, followed by elution of the final library in 15 μL (0.1X TE buffer) (Clevergene Biocorp Private Limited, Bangalore).

### 2.6 Library quantification

The DNA BR assay reagent stands out as an exceptionally sensitive detection dye for precisely quantifying DNA or library content in a solution. It offers a linear detection of fluorescence range from 100 pg./μL to 1,000 ng/μL. A ratio of 1:200 was utilized for diluting dye and buffer, followed by the addition of a library (1 μL). Furthermore, incubation was carried out for 2 min at room temperature, and subsequently, measurements were recorded by Qubit 3 Fluorometer (Life technologies/Catalog: Q33216). Prior to measurement, calibration was performed by the standards provided in the kit. The instrument was calibrated with the two standards supplied in the kit before measuring the sample.

### 2.7 Sequencing methodology

A total of 25 ng DNA was utilized for amplification of the (V3-V4) hypervariable region. The reaction involves modified primers (341F and 785R) of 100 nm and KAPA HiFi HotStart Ready Mix (Klindworth et al., 2013). PCR steps were similarly followed as described in Section 2.5. Ampure beads were used to remove unwanted primers. Furthermore, using Illumina barcoded adapters, 8 cycles of PCR were performed to prepare the sequencing libraries of 16S rRNA (V3–V4). Amplification of 16S bacterial rRNA gene was carried out using PCR targeting the V3-V4 regions using the following primers: (forward: 5′ CCTACGGGNGGCWGCAG; and reverse: GACTACHVGGGTATCTAATCC). A library was constructed by targeting the 16S V3–V4 regions, and sequencing was performed using the Illumina MiSeq platform with 300 bp paired-end libraries. The sequence data were produced using the Illumina MiSeq platform, and its quality was assessed using FastQC (Edgar et al., 2011) and MultiQC (DeSantis et al., 2006) software.

## 3 Bioinformatic analysis

### 3.1 Operational taxonomic units and statistical analyses

The sequences obtained from PCR were amplified and processed for quality inspection. Furthermore, reads were filtered using Trim Galore^1^ via the Cutadapt tool. UCHIME algorithm (Edgar et al., 2011) was applied to flag contigs containing chimeric regions. Using the GREENGENES v.13.8-99 database, the filtered contigs were processed and classified into taxonomical categories (DeSantis et al., 2006). Operational taxonomic units (OTUs) were obtained after clustering the contigs followed by estimation of OTU abundance. Mothur pipeline (Schloss et al., 2009) was used to determine the alpha and beta diversity indices. Phylum, genus, and species level taxonomical analysis was plotted using the PAST 4.03 tool and GraphPad Prism statistical software. A *p*-value of < 0.05 was regarded as statistically significant using one-way ANOVA with *post-hoc* Dunnett’s multiple comparisons test.

### 3.2 Metagenomic data analysis

For the removal of the degenerate primers, reads (20 bp) were processed by trimming from the 5′ end, followed by the elimination of low-quality bases and adapter sequences using Trim Galore. Reads that passed the quality control test were streamlined into Mothur (Schloss et al., 2009), making the aligned pairs form contigs. The contigs whose size falls within the range of 300–532 bp were reserved after the process of error screening. Furthermore, high-quality contigs were quantified for similar sequences while any ambiguous base calls were rejected. The contigs were then aligned to a standard database for 16S rRNA. Despite the use of pre-designed 16S bacterial primers, chances of non-specific amplification exist. Most of the contigs were aligned to their specific target databases. Based on the amplification of variable regions, any ambiguous contigs aligned to non-specific sites on the database were removed. Then followed by the removal of overhangs and gaps at their end sites from the contigs to be processed for the removal of chimera, which are raised due to PCR errors. Later, the UCHIME algorithm (Edgar et al., 2011) was availed to flag contigs with chimeric regions. Standard references for all the chimeric sequences were utilized to recognize and eliminate possible chimeric sequences. GREENGENES v.13.8–99 database was additionally used to classify OTUs after processing the filtered contigs (DeSantis et al., 2006). Finally, for the final estimation of abundance, contigs were clustered into OTUs (Supplementary file S1).

## 4 Results

### 4.1 Gut microbiota richness and diversity analysis

**Figure 2:**
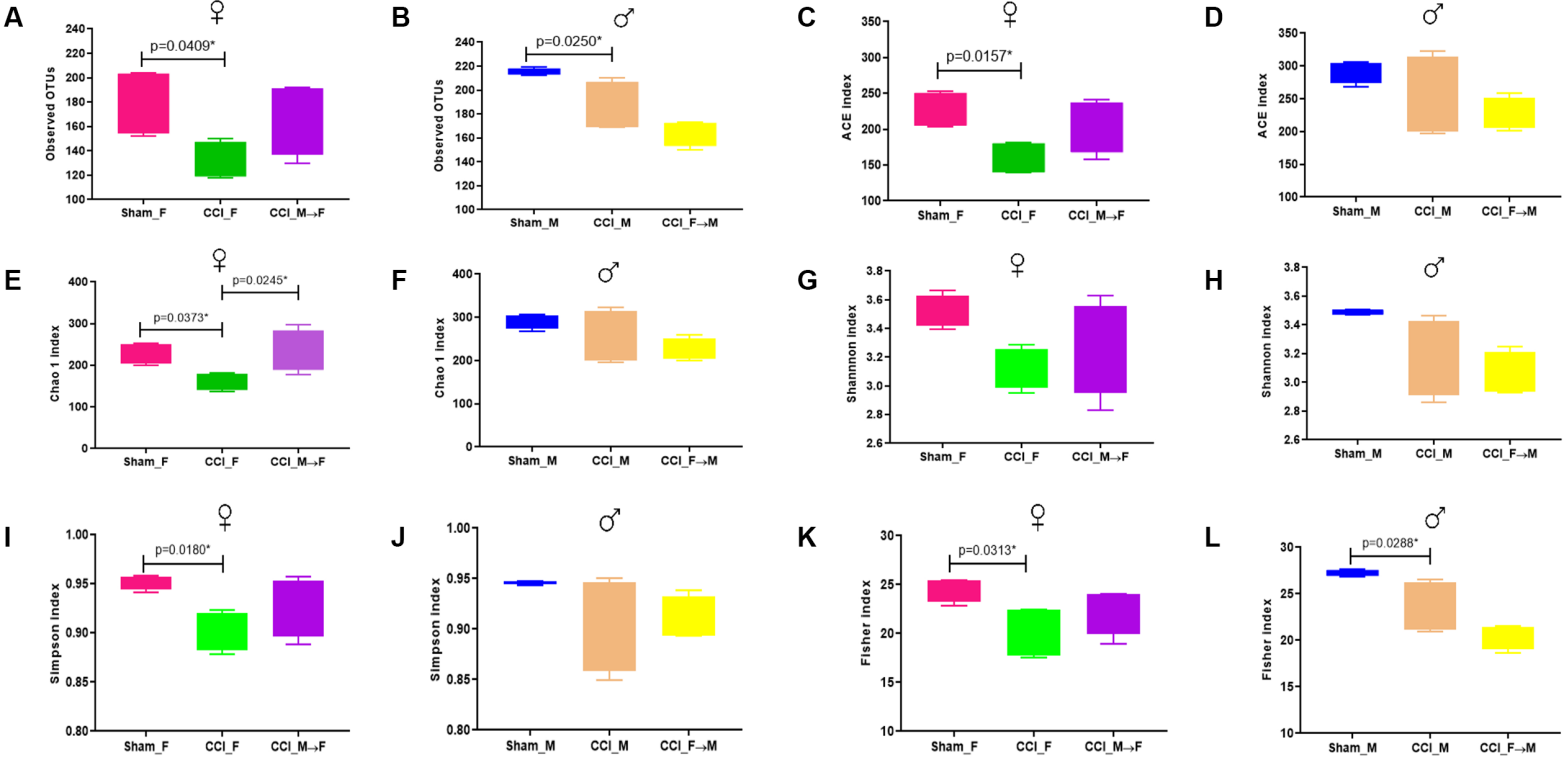
Graphs showing the richness and diversity index. Observed OTUs were **(A)** *p* = 0.0409 vs. CCI_F and **(B)** *p* = 0.0250 vs. CCI_M mice. ACE index showing *p* = 0.0157 vs. CCI_F mice **(C,D)**. No significant difference compared with CCI_M mice. Chao 1 index **(E)** *p* = 0.0373 vs. CCI_F, and no significant difference was seen in CCI_M mice **(F)**. Shannon index **(G,H)** represents a greater decline in CCI mice of both sexes compared with Sham and FMT groups. Simpson index showing *p* = 0.0180 vs. CCI_F **(I)**, and no significant difference is seen in CCI_M **(J)**. Fisher index **(K,L)** represents *p* = 0.0313 vs. CCI_F and *p* = 0.0288 vs. CCI_M mice, respectively.

The alpha diversity indices of gut microbiota were evaluated at 3 dpi using a 16S rRNA gene amplicon (Figures 2A–L; Supplementary file S2). One-way ANOVA revealed a reduced microbiota richness in both male and female mice after CCI (CCI_M and CCI_F) compared with sham-operated mice, as indicated by observed OTUs, ACE, and Chao 1 index. Moreover, reduced abundance and evenness (Shannon, Simpson, and Fisher indices) of gut microbes were observed in CCI_M and CCI_F mice. These findings were also evident in a few earlier studies (Du et al., 2021; Hou et al., 2021).

In the FMT module, we noticed that female mice receiving microbiota from male donors (CCI_M → F) had a higher OTU score than male mice who received microbiota from female donors (CCI_F → M). Assessing the gut microbiota richness using the ACE and Chao 1 indices revealed a notable increase in richness in CCI_M → F mice compared with CCI_F → M mice. Furthermore, CCI_M → F mice also demonstrated the greater evenness and abundance, as indicated by Shannon, Simpson, and Fisher indices, than both male and female sham-operated mice.

**Figure 3:**
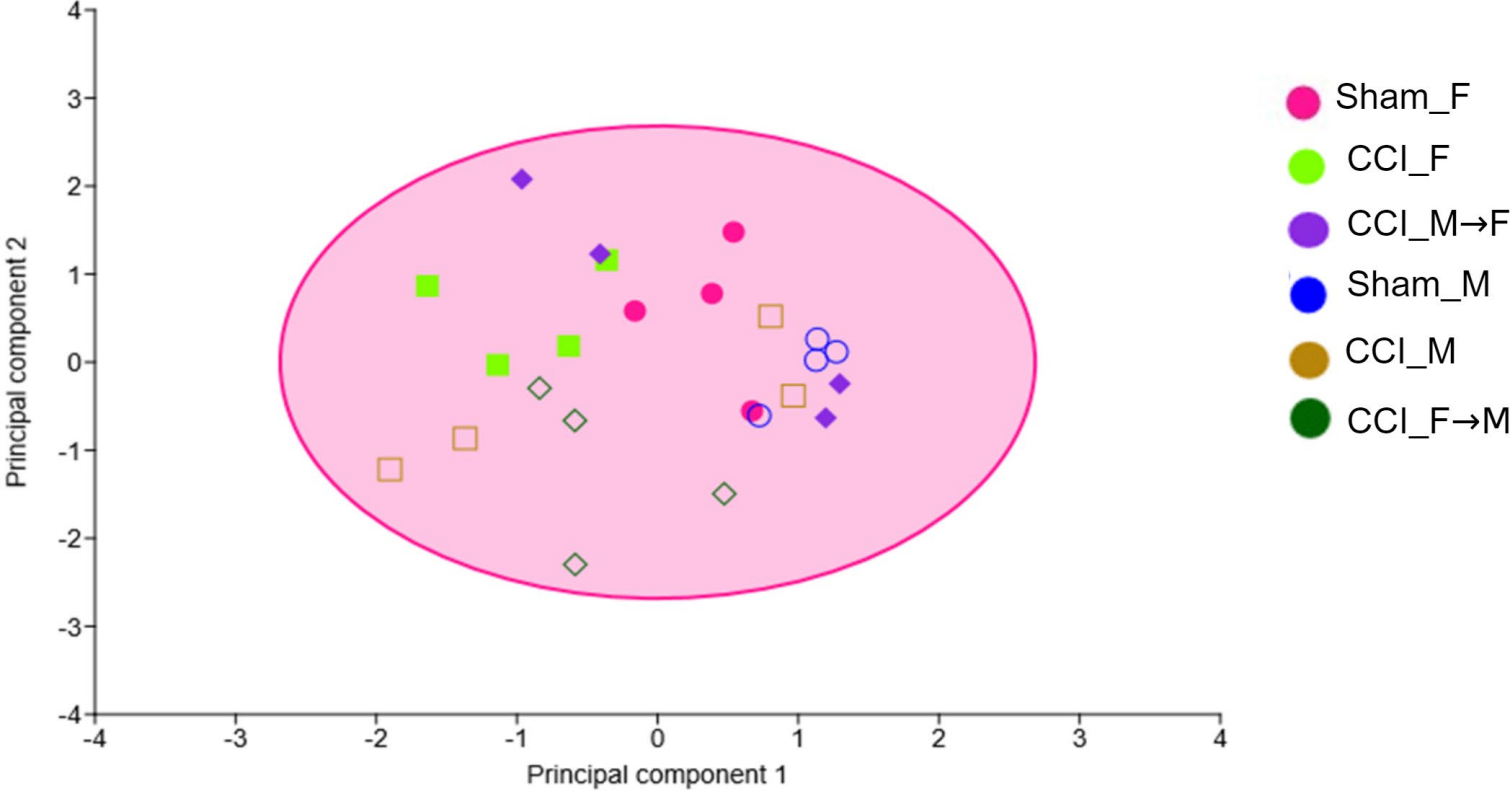
The graph showing the principal component analysis (PCA) of the fecal samples at 3 dpi. Symbols with different colors and shapes represent different groups (six).

Gut microbiota beta diversity (Supplementary file S3) was assessed by analyzing OTU-level differences across the different groups. The dissimilarities index was calculated on a variance and co-variance matrix using a 4-point eigenvalue scale and represented in a principal component analysis (PCA) plot using PAST 4.03 (Figure 3). The plot showed minimal dissimilarities between Sham_F vs. CCI_F and Sham_M vs. CCI_M groups. Similarly, no major beta diversity was seen in male and female mice after CCI at 3 dpi. In fecal-engrafted groups also, we did not observe major sex-specific differences in OTU diversity.

### 4.2 Phylum level changes in gut microbiota

**Figure 4:**
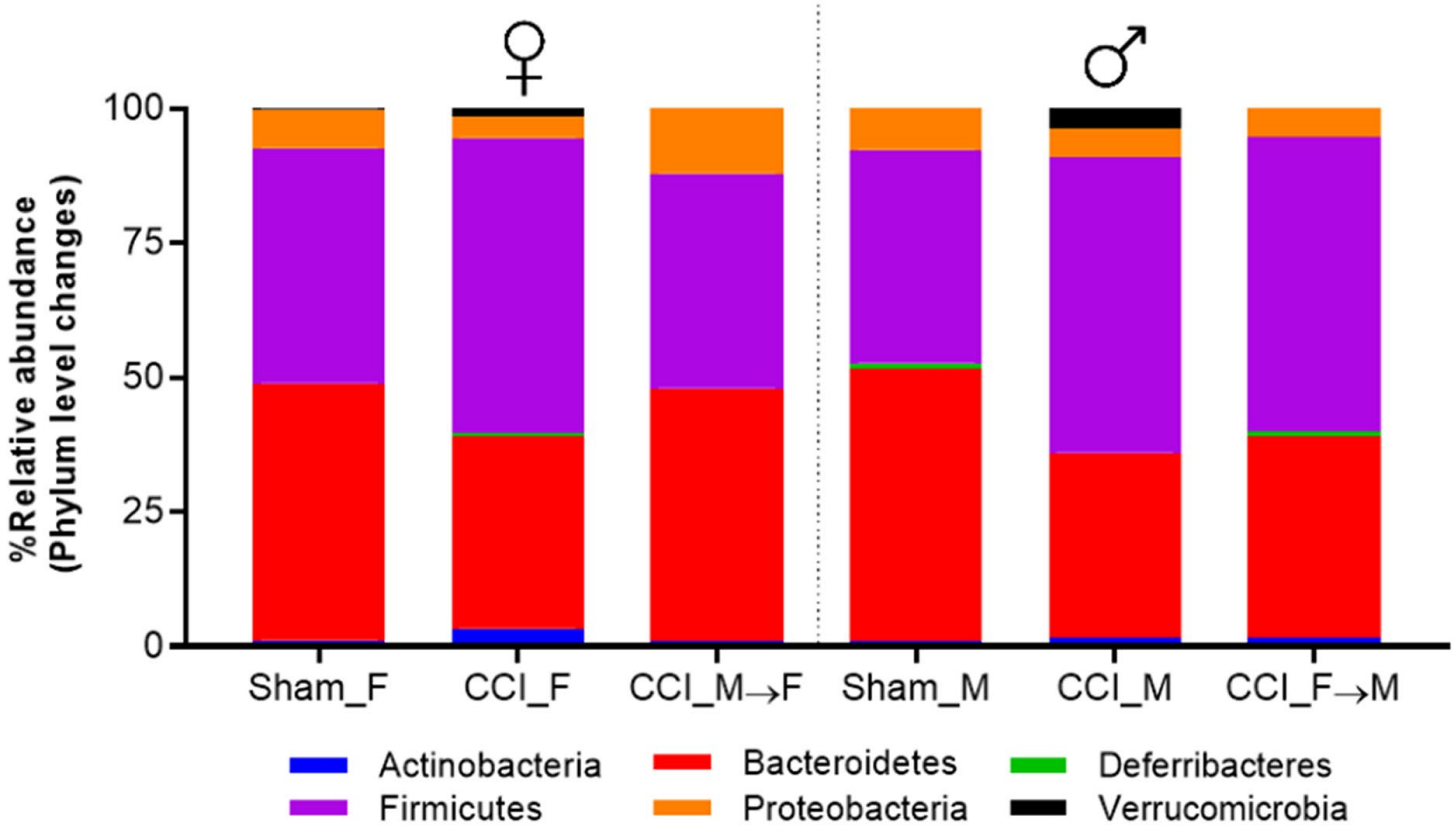
Graph representing the percentage relative abundance at top 6 phyla across the Sham male and female mice, CCI-induced female (CCI_F) and male (CCI_M) mice and fecal microbial recipient groups, i.e., CCI_M → F (male mice as a donor to female mice as the recipient) and CCI_F → M (Female mice as a donor to male mice as the recipient) using GraphPad Prism software.

**Figure 5:**
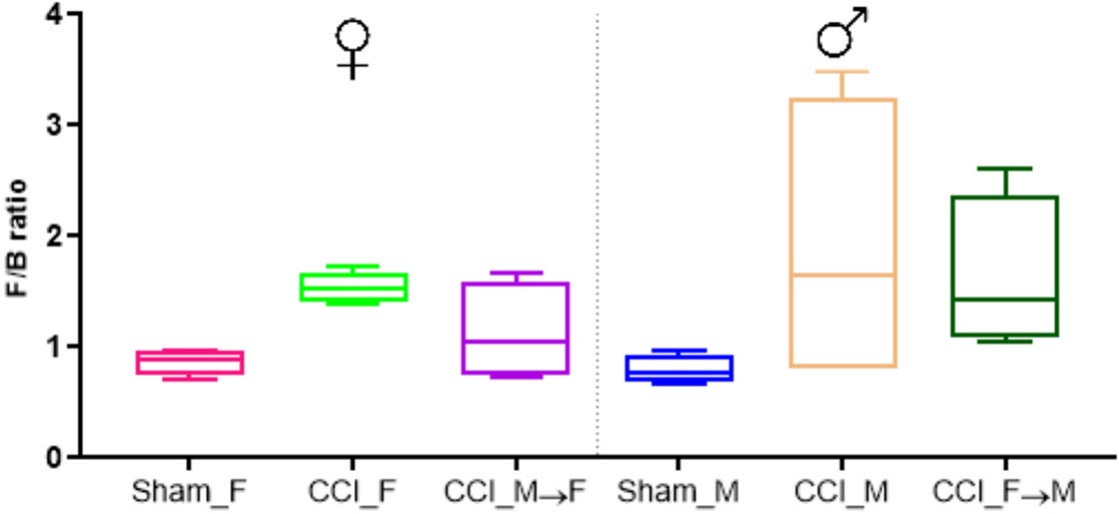
Graph representing the *Firmicutes* to *Bacteroidetes* (F/B) ratio across the sham mice of female and male (Sham_F and Sham_M), CCI-induced female (CCI_F), and male (CCI_M) mice and fecal microbial recipient groups, i.e., CCI_M → F (male mice as donor to female mice as recipient) and CCI_F → M (Female mice as a donor to male mice as recipient) using GraphPad Prism software.

The phylum-level changes in the gut microbiota phenotypes are depicted in a stacked bar chart (Figure 4, Supplementary file S4). The most abundant phyla in Sham_F constitute *Bacteroidetes* (47%), *Firmicutes* (43.65%), *Proteobacteria* (7.2%), and others (1.44%). The compositional distribution of Sham_M mice was *Bacteroidetes* (50.75%), *Firmicutes* (39.70%), *Proteobacteria* (7.5%), and others (1.96%). At 3 dpi, CCI_F and CCI_M mice displayed a decline in the abundance of *Bacteroidetes*. Furthermore, changes in the phylum abundance were also noticed in FMT-recipient mice. Specifically, among CCI_M → F mice, there was an increase in *Bacteroidetes* (46.8%) and a decrease in *Firmicutes* (39.9%) composition. Conversely, CCI_F → M mice exhibited the opposite pattern with a decline in *Bacteroidetes* (37.4%) and a rise in *Firmicutes* (54.7%). In the *Firmicutes/Bacteroidetes* (F/B) ratio (Figure 5), the increased F/B ratio was evident in CCI_M and CCI_F mice, signifying gut dysbiosis; additionally, in fecal-engrafted mice, a ratio of 1.1 (CCI_M → F mice) and 1.6 (CCI_F → M) was noticed.

### 4.3 Genus level changes in gut microbiota

Analysis of fecal gut microbiome samples through polar plots in PAST 4.03 unveiled a rapid shift in the abundance of the genera at 3 dpi such as *Akkermansia*, *Alistipes*, *Bacteroides*, *Bifidobacterium*, *Clostridium*, *Lactobacillus*, *Prevotella*, and *Ruminococcus* in CCI_M and CCI_F mice, as well as in FMT groups. Unlike CCI_M mice, a notable decline in the levels of *Alistipes*, *Lactobacillus*, and *Prevotella* genera was observed in the CCI_F group compared with sham. Furthermore, a noticeable increase in the relative prevalence of *Akkermansia* and *Ruminococcus* genera was also seen in CCI_M and CCI_F mice of both sexes. Considering the significant shifts in gut microbial communities, it is worth mentioning that the CCI_F group exhibited a marked reduction in the *Lactobacillus* genus compared with the sham-operated female mice.

**Figure 6:**
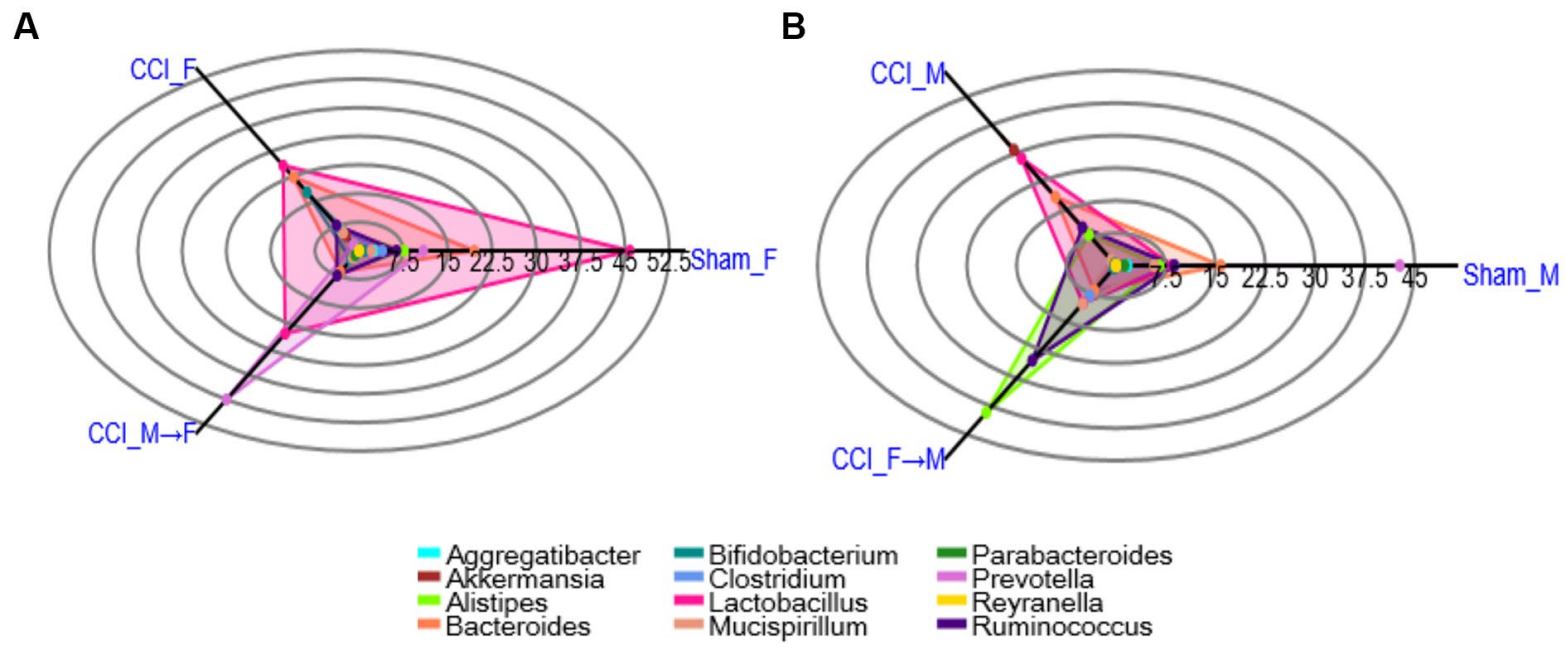
Polar plots showing the abundance of top 12 genus level across the Female **(A)** and Male **(B)** mice. Groups representing: sham mice of female and male (Sham_F and Sham_M), CCI induced female (CCI_F) and male (CCI_M) mice and fecal microbial recipient groups i.e., CCI_M→F (male mice as donor to female mice as recipient) and CCI_F→M (Female mice as a donor to male mice as recipient) using PAST 4.03 software.

The examination of sex-specific changes in gut microbiota using the FMT approach has revealed a notable change in *Lactobacillus* genus in CCI_M → F group as compared to that in CCI_F → M (Figure 6; Supplementary file S5). Marked elevation of *prevotella* was noticed in the CCI_M → F group, while the increase in *Alistipes* and *Ruminococcus* was seen in CCI_F → M engrafted mice. Furthermore, when examining the abundance in sham mice of both sexes, no notable changes have been observed. However, sham_F mice exhibited a higher percentage of *Lactobacillus* abundance than sham_M mice.

### 4.4 Species-level changes in gut microbiota

**Figure 7:**
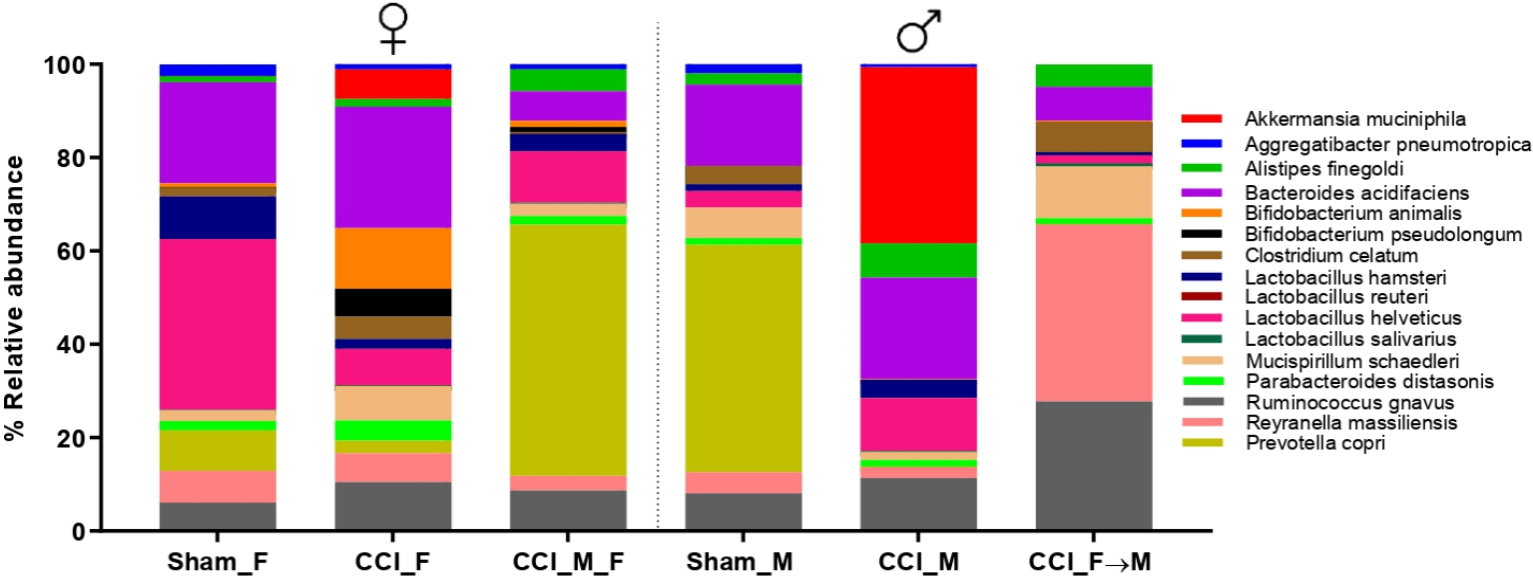
Stacked bar plot showing the abundance of top 16 species level across the sham mice of female and male (Sham_F and Sham_M), CCI-induced female (CCI_F) and male (CCI_M) mice, and fecal microbial recipient groups, i.e., CCI_M → F (male mice as donor to female mice as recipient) and CCI_F → M (Female mice as a donor to male mice as recipient) using GraphPad Prism software.

**Figure 8:**
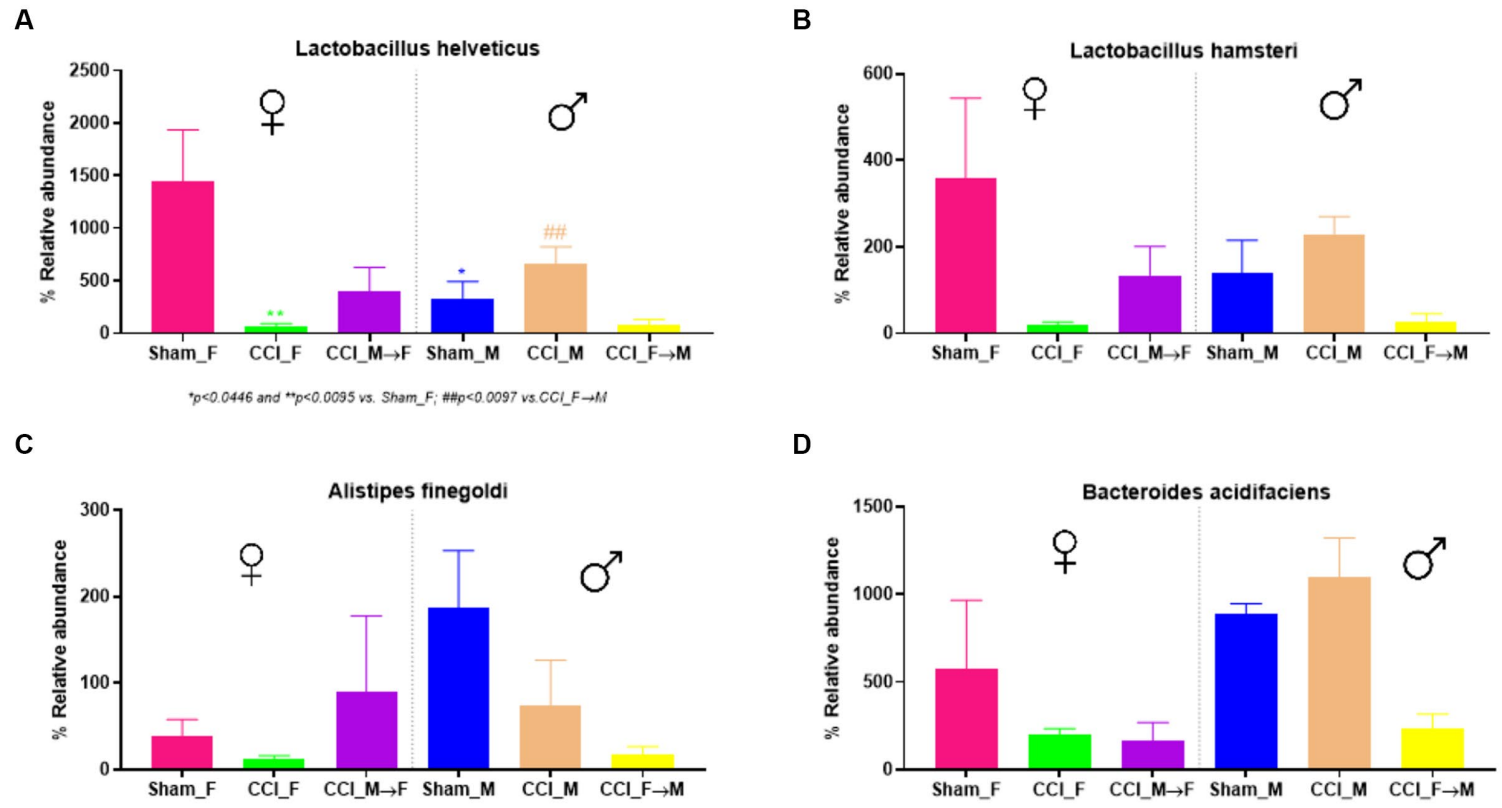
Graphs showing the abundance of *Lactobacillus helveticus* **(A)**, *Lactobacillus hamsteri* **(B)**, *Alistipes finegoldii* **(C)**, and *Bacteroides acidifaciens* **(D)** using GraphPad Prism software. The significance of *p* = 0.0095 vs. CCI_F was noticed in female mice.

Sham_F mice displayed a greater abundance of specific species (*Bacteroides* and *Lactobacillus*) in contrast to sham_M mice, as depicted in the stacked plot (Figure 7). At 3 dpi, a rapid shift in the abundance of *Akkermansia muciniphila*, *Alistipes finegoldii*, *Bacteroides acidifaciens*, *L. hamsteri*, *L. helveticus*, *Prevotella copri*, and *Ruminococcus gnavus* was observed at 3 dpi in both sexes as well as in FMT groups. In contrast to CCI_M mice, CCI_F mice displayed a marked decline in the abundance of *L. helveticus* and *L. hamsteri*. A significantly decreased abundance of *L. helveticus* was observed in CCI_F mice (*p* = 0.0095 vs. sham_F) as compared to CCI_M mice (Figure 8A). Furthermore, *L. hamsteri* showed the reduced abundance in CCI_F mice compared to CCI_M mice, but this change was not significant statistically when compared to sham_F mice (Figure 8B). One-way ANOVA followed by *post-hoc* Tukey’s multiple comparisons test revealed a decline in the abundance of *Alistipes finegoldii* and *Bacteroides acidifaciens* (Figures 8C,D), with no major statistical significance.

**Figure 9:**
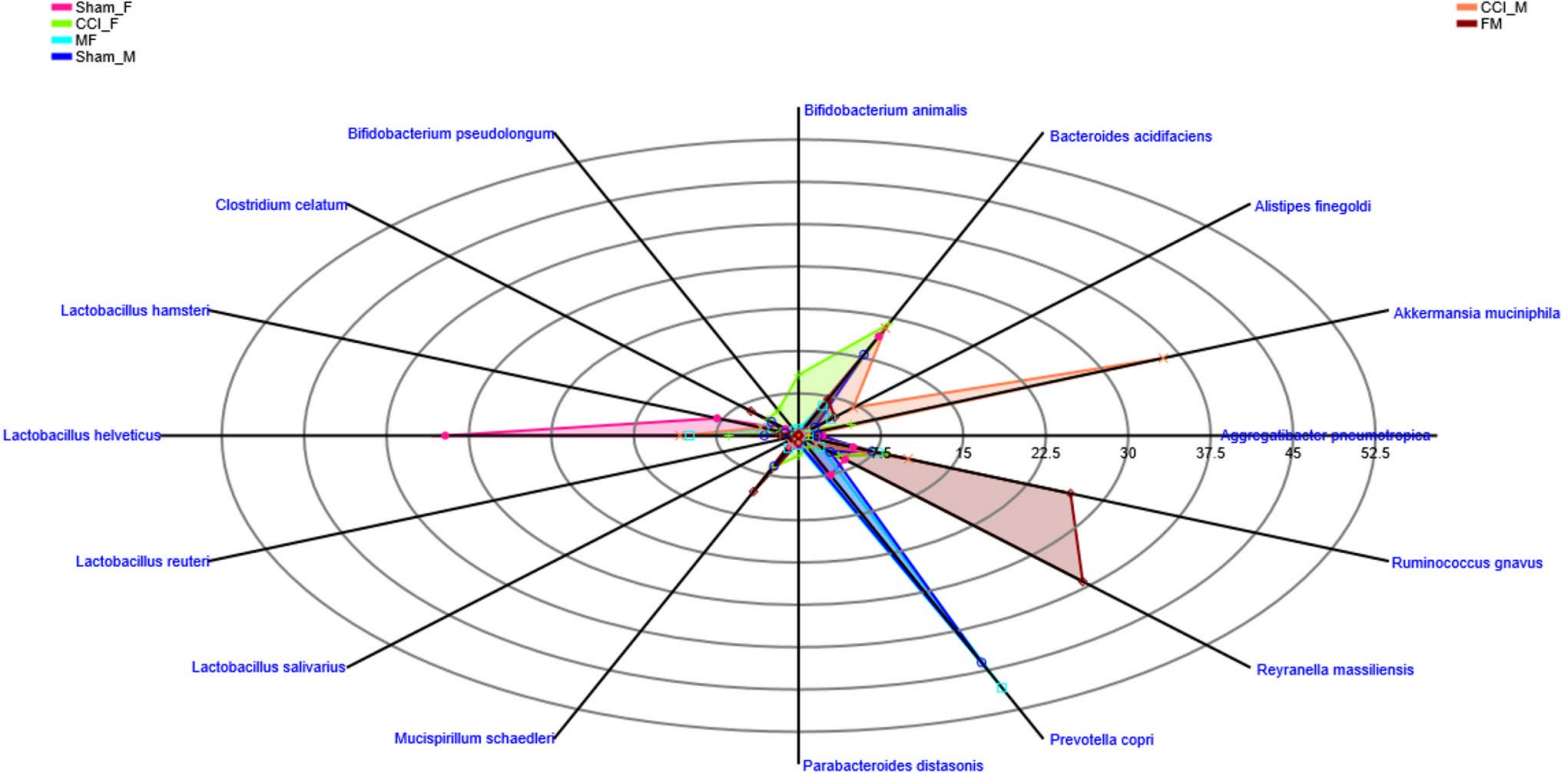
Radar plot representing the sex-specific percentage distribution of species among all the groups showing the greater richness and abundance across 16 represented species using PAST 4.03 software.

Radar plots were employed to depict the sex-specific changes at the species level (Figure 9). These plots represented a greater richness and abundance in *L. helveticus* and *L. hamsteri*. Notably, a greater richness of these *Lactobacillus* species was noticed in CCI_M → F group as compared to CCI_F → M. These findings underscore the significance of *Lactobacillus* in potential therapeutic interventions (Supplementary file S6).

**Figure 10:**
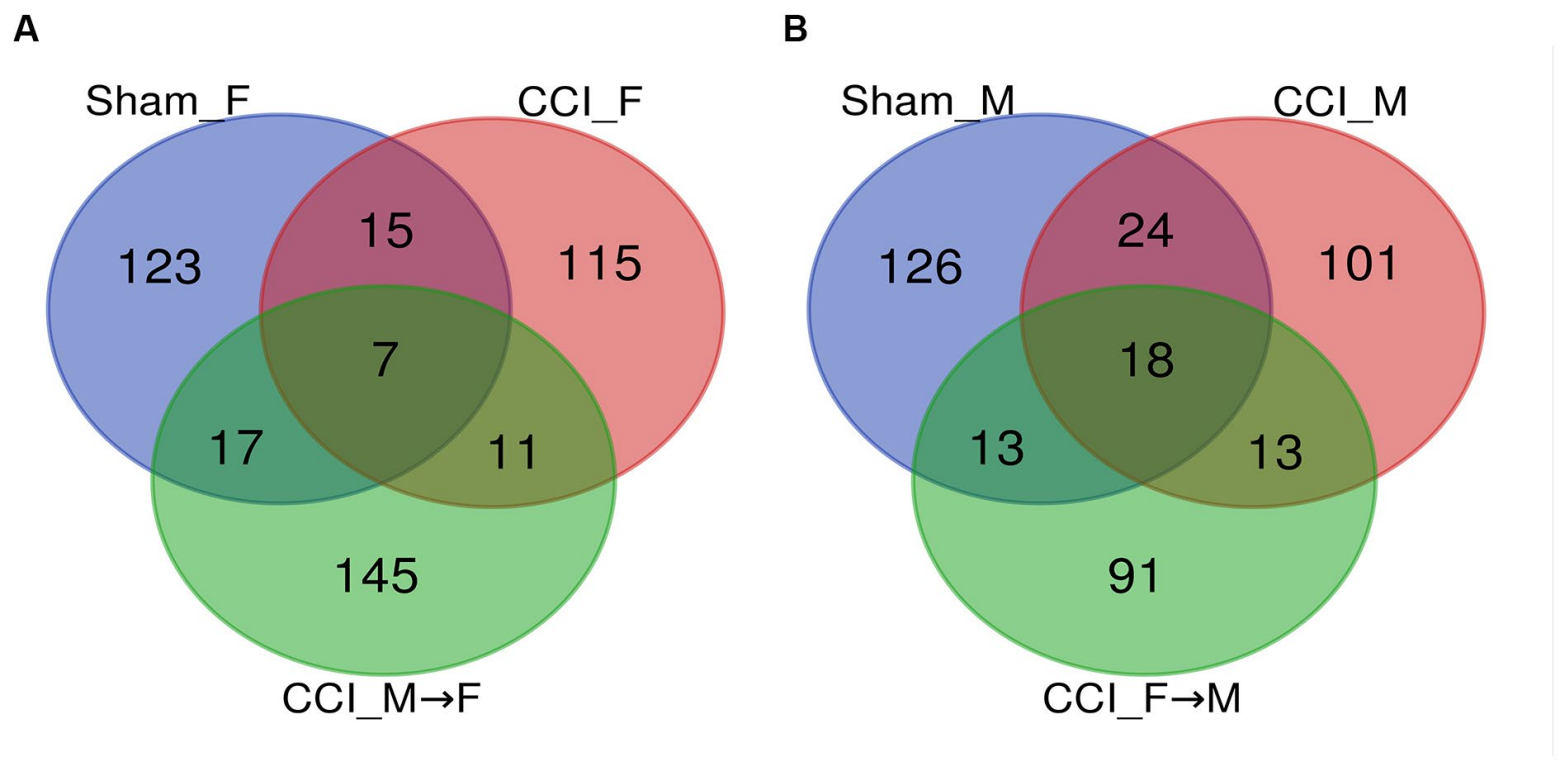
Venn diagram representing bacterial community OTUs (genus and species) distribution, and intersection among the female **(A)** and male mice **(B)**.

**Figure 11:**
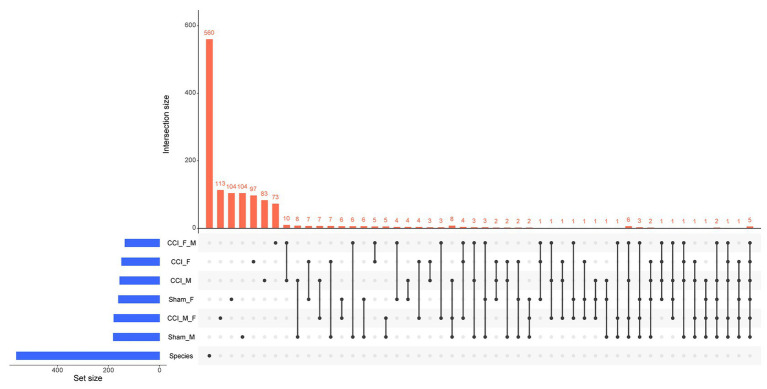
UpSet plot eliciting the intersection of species among different groups. The total size of each set is represented on the left bar plot. Every connection is represented by the bottom plot, and their occurrence is shown on the top bar plot.

A Venn diagram (Figures 10A,B) illustrated the occupancy and distribution of the core microbiome of 568 OTUs among six groups. The Venn plot (Supplementary file S7) also revealed the combined OTUs among groups such as sham_F vs. CCI_F vs. CCI_M → F (Ma et al., 2017) and sham_M vs. CCI_M vs. CCI_F → M (Evans et al., 2015). The UpSet plot (Supplementary file S8) displayed the interaction size of genera and species among six groups (Figure 11). It was observed that 104, 97, 113, 104, 83, and 73 OTUs were unique to sham_F, CCI_F, CCI_M → F, sham_M, CCI_M, and CCI_F → M groups, respectively, and five OTUs were commonly shared across all the groups.

## 5 Discussion

A key impact of gut microbiota has been demonstrated in the regulation of inflammation, immune responses, anxiety-related outcomes, and post-injury consequences (Kelly et al., 2016; Robertson et al., 2017; Davis et al., 2022; Medel-Matus et al., 2022). Numerous studies also emphasized the importance of gut microbiota in post-TBI complications (Hang et al., 2003; Ma et al., 2017; Nicholson et al., 2019; Du et al., 2021). The CCI surgery in rodents mimics several clinical symptoms of the contusion type of TBI and also displays a wide change in gut microbiota phenotypes (Evans et al., 2015; Hazeldine et al., 2015; Treangen et al., 2018; Zhu et al., 2018). In this study, we chose a 3-dpi time point for the investigation of phylum, genus, and species level changes in fecal samples, as it is a critical time point with respect to hippocampal neurogenesis and histopathological changes in the brain after CCI (Sun et al., 2007; Kernie and Parent, 2010; Pabón et al., 2016). We observed a substantial decrease in bacterial richness (ACE and Chao 1 index) after CCI in both male and female mice. Moreover, reduced evenness and abundance of gut microbiota were reflected in Shannon, Simpson, and Fisher indices in both male and female mice. These alpha diversity-related changes in gut microbiota following CCI correspond with the earlier research findings (Nicholson et al., 2019; Davis et al., 2021, 2022; Du et al., 2021; Frankot et al., 2023). The bidirectional crosstalk of the brain and gut microbiota has also been evident in the management of brain injuries such as stroke, spinal cord injuries, and TBI (Singh et al., 2016; Akhoundzadeh et al., 2018; Sun et al., 2018; Ma et al., 2019; Devi et al., 2021; Frankot et al., 2023). Thus, as suggested in earlier studies (Benakis et al., 2016; Ma et al., 2019), rebuilding gut microflora biodiversity may be essential for recovery from brain injury.

In this study, CCI-operated male and female mice showed noticeable changes in the genera of *Akkermansia*, *Alistipes*, *Bacteroides*, *Bifidobacterium*, *Clostridium*, *Lactobacillus*, *Prevotella*, and *Ruminococcus* at 3 dpi. The *Alistipes* genus is abundantly found in human gut microbiota (Hou et al., 2021), which offers dimorphic effects, i.e., protection against colitis and autism spectrum disorder, and also potentially contributes to anxiety, myalgic encephalomyelitis/chronic fatigue syndrome, and depression (Koren et al., 2013; Morgan et al., 2013; Manrique et al., 2016; Parker et al., 2020). *Ruminococcus* bacterium plays a key role in stress management in wild and domestic animals (Rowin et al., 2017). In line with earlier TBI studies, an increase in *Ruminococcus* species in both male and female CCI mice was noticed (Celorrio et al., 2021). Recent investigations on *Lactobacillus* and *Bifidobacterium* containing probiotics illustrated the role of these bacteria in the reversal of anxiety, chronic psychological stress, neuronal death, and cognitive dysfunction in mice (Messaoudi et al., 2011a,b; Zakostelska et al., 2011).

We noted a greater abundance of *Akkermansia* at 3 dpi in male mice than in female mice. The increased abundance of *Akkermansia* has already been indicated in TBI patients and brain injury rat models (Hou et al., 2021). The variable levels of *Clostridium* species were seen in both male and female mice after CCI, which influences inflammatory bowel disease (Kabeerdoss et al., 2013; Lopetuso et al., 2013). Moreover, we noted differences in *P. copri* abundance, which are connected with elevated serum amino acids, especially branched-chain amino acids (Pedersen et al., 2016). The relevance of *P. copri* has been correlated with bacterial vaginosis, rheumatoid arthritis, periodontitis, and other chronic inflammatory states (Pianta et al., 2017; Accetto and Avguštin, 2021). We also noted the increased abundance of *P. copri* in the CCI_M → F group. In this way, sex-specific characterization of gut microbiota phenotypes may be useful for predicting post-TBI neurological recovery and crafting better therapeutic approaches (Luczynski et al., 2016; Sundman et al., 2017; Davis et al., 2021; Du et al., 2021).

TBI reduces gut microbial diversity and disrupts the ratio of healthy to opportunistic bacteria. Our 16S rRNA gene amplicon results revealed a substantial decline in microbial diversity. We found a notable change in the diversity of *Lactobacillus* genera, indicating the therapeutic importance of *Lactobacillus*. The *Lactobacillus* genus was decreased in CCI_F compared with CCI_M mice. Moreover, the abundance of *L. helveticus* and *L. hamsteri* has indicated that specific beneficial gut microbiota may play a pivotal lead in the recovery process following a brain injury. Furthermore, the potential neuroprotective benefits of probiotics have already been reported for neurotraumatic events (Fransen et al., 2017). For instance, *L. acidophilus* and *L. reuteri* improve post-TBI neuronal complications and brain edema by increasing the expression of occludin and neuronal survival (Deitch et al., 2007; Lavoie et al., 2017). These findings provide promising avenues for better therapeutic strategies in treating neurological illnesses (Messaoudi et al., 2011a,b; Zakostelska et al., 2011). As probiotics impose a positive impact on brain functions, they also possess a role in influencing the diversity of the gut microbiota, which is crucial for overall wellbeing (Bienenstock et al., 2015; Sirisinha, 2016; Akhoundzadeh et al., 2018; Ma et al., 2019; Devi et al., 2021; Savigamin et al., 2022). These preliminary results indicate that the sex-specific restoration of gut microbiota may reverse the CCI-generated neurological complications.

## 6 Conclusion

In this study, we observed an increase in the *Alistipes* and *Bacteroidetes* genera following CCI surgery in both male and female mice. The sex-specific changes were identified in the *P. copri*, *L. helveticus*, and *L. hamsteri* species at 3 dpi. We suggest that the modulation of these microbial strains may hold promise for developing sex-specific gut microbiota-based interventions in cases of brain injury.

## Data availability statement

The datasets presented in this study can be found in online repositories. The names of the repository/repositories and accession number(s) SRA (Sequence Read Archive) files were submitted to NCBI with accession number BioSample accession: SAMN38196246 and Bioproject ID: PRJNA1037720) can be found in the article/Supplementary material.

## Ethics statement

The animal studies were approved by Institutional Animal Ethics Committee of the NIPER, Hyderabad (Approval number: NIP/10/2020/PC/377). The studies were conducted in accordance with the local legislation and institutional requirements. Written informed consent was obtained from the owners for the participation of their animals in this study.

## Author contributions

TP: Data curation, Formal analysis, Writing – original draft, Writing – review & editing. MD: Supervision, Writing – review & editing.

## Footnotes

^1^ http://www.bioinformatics.babraham.ac.uk/projects/trim_galore/
